# Epigenetic Influence of Dam Methylation on Gene Expression and Attachment in Uropathogenic *Escherichia coli*

**DOI:** 10.3389/fpubh.2016.00131

**Published:** 2016-06-27

**Authors:** Stacy Ann-Marie Stephenson, Paul D. Brown

**Affiliations:** ^1^Department of Basic Medical Sciences, Faculty of Medical Sciences, University of West Indies, Jamaica

**Keywords:** dam methylation, uropathogenic *Escherichia coli*, λ red recombineering, gene expression, fluoroquinolone resistance

## Abstract

Urinary tract infections (UTI) are among the most frequently encountered infections in clinical practice globally. Predominantly a burden among female adults and infants, UTIs primarily caused by uropathogenic *Escherichia coli* (UPEC) results in high morbidity and fiscal health strains. During pathogenesis, colonization of the urinary tract *via* fimbrial adhesion to mucosal cells is the most critical point in infection and has been linked to DNA methylation. Furthermore, with continuous exposure to antibiotics as the standard therapeutic strategy, UPEC has evolved to become highly adaptable in circumventing the effect of antimicrobial agents and host defenses. Hence, the need for alternative treatment strategies arises. Since differential DNA methylation is observed as a critical precursor to virulence in various pathogenic bacteria, this body of work sought to assess the influence of the DNA adenine methylase (*dam*) gene on gene expression and cellular adhesion in UPEC and its potential as a therapeutic target. To monitor the influence of *dam* on attachment and FQ resistance, selected UPEC *dam* mutants created *via* one-step allelic exchange were transformed with cloned *qnr*A and *dam* complement plasmid for comparative analysis of growth rate, antimicrobial susceptibility, biofilm formation, gene expression, and mammalian cell attachment. The absence of DNA methylation among *dam* mutants was apparent. Varying deficiencies in cell growth, antimicrobial resistance and biofilm formation, alongside low-level increases in gene expression (*rec*A and p*ap*I), and adherence to HEK-293 and HTB-9 mammalian cells were also detected as a factor of SOS induction to result in increased mutability. Phenotypic characteristics of parental strains were restored in *dam* complement strains. *Dam*’s vital role in DNA methylation and gene expression in local UPEC isolates was confirmed. Similarly to *dam*-deficient Enterohemorrhagic *E. coli* (EHEC), these findings suggest unsuccessful therapeutic use of Dam inhibitors against UPEC or *dam*-deficient UPEC strains as attenuated live vaccines. However, further investigations are necessary to determine the post-transcriptional influence of *dam* on the regulatory network of virulence genes central to pathogenesis.

## Introduction

Uropathogenic *Escherichia coli* (UPEC) is the most ubiquitous pathogen implicated in urinary tract infections (UTIs), accounting for 80–90% of all infections (1, 2). Numerous studies have reported the prevalence of various virulence factors among UPEC isolated from the urinary tract including the adhesins (Type 1, P, S, and F1C fimbriae), toxins (RTX and CNF-1), pore-forming hemolysin, aerobactin, and multiple sideophore-based iron acquisition systems (3–7). Several publications report the existence of these virulence factors in clusters, small virulence cassettes, or large blocks of genes in enteropathogenic, enterohaemorragic, and UPEC strains, not observed in the genome of fecal isolates (3, 8–14). These factors are often linked or co-regulated, acting in concert according to host response and environmental signals (15). Such features contribute to the adherence, colonization, multiplicity, propagation, and persistence of UPEC strains within the mammalian host’s urinary tract, despite hydrodynamic challenges encountered (16).

The adhesion of UPEC to mucosal cells is the most vital step for the initiation of UTI and is mediated by P and Type 1 fimbriae, binding to digalactoside-containing globoseries glycosphingolipids and mono d-mannose residues, respectively (17–21). However, this report focuses on the vastly studied, mannose-resistant *pap* (pyelonephritis-associated pili) fimbriae by which genotypic studies have identified the gene in approximately 80% of *E. coli* isolates that cause pyelonephritis (15). The *pap* operon mainly consists of fimbriae structural subunits (*pap*A, -C, -D, -E, -F, -G); subunit to terminate fimbrial growth and anchor mature fimbriae to host cell surface, *pap*H; and the divergently encoded regulatory genes *pap*B and *pap*I, within which the main promoter is located. Expression of these promoters is dependent on the methylation status of the two GATC sites within the intergenic region (19, 22). Although there are reports that support fimbriae-mediated UPEC adherence *in vitro* (7, 23, 24), the significance of P fimbriae for infectivity has not been confirmed as only subtle adherence roles were exhibited in uroepithelial cell culture models (25).

DNA methylation is a vital epigenetic, postreplicative alteration that is catalyzed by a class of enzymes referred to as the DNA methyltransferases (MTases). Crucial to the regulation of many cellular processes in eukaryotes and prokaryotes, DNA adenine methylase (Dam) plays numerous roles in DNA mismatch repair, transcriptional regulation, and SOS response stimulation as part of the cell cycle (26–30). In *E. coli*, the *Dam* enzyme catalyzes the postreplicative transfer of methyl from *S*-adenosyl-l-methionine to the N-6 position of adenine in the tetranucleotide GATC sequence in hemimethylated DNA (28, 31–34). Conserved for the manifestation of virulent genes, *Dam*’s essential function in pathogenesis has also been reported in several bacterial species including *E. coli, Salmonella enterica* serovar Typhimurium, *Yersinia* spp*., Haemophilus influenzae, Vibrio cholera*, and *Pasterella multocidas* (28, 31, 32, 35). The presence of GATC sites in the −10 and −35 hexamers of promotor regions, directly affecting gene expression by regulating the binding of transcriptional factors or RNA polymerase according to methylation state at the sites, serves to substantiate this phenomenon.

Though not essential to the viability of *E. coli*, Dam’s involvement in transcriptional modulation was first proven through detailed studies of *pap* operon encoding pili necessary for UTI in UPEC (26, 28, 31, 33, 35–40). The *pap* pili-mediating adhesion of UPEC to mammalian uroepithelial cells is epigenetically regulated through methylation at the intergenic region of *pap*IB to produce “ON/OFF” phase variation (expression or non-expression, respectively) by individual cells (30, 34, 38, 41). The phase variation mechanism *via* Dam methylation pattern acts a switch for the expression of the operon and confirms reports for the significance of Lrp, PapI, and Dam as transcription regulators (28, 31, 34, 38, 42, 43). The reversible expression of *pap* is theorized to allow the bacteria to attach and detach from the urogenital tissues, which in turn enables colonization and infection (33).

Previous research indicates Dam-deficient (Dam^−^) mutants of pathogenic *Salmonella* serovar Typhimurium being rendered avirulent with the overexpression of over 35 genes (44) and downregulation of others, preferentially expressed during infection. This was made evident by Badie et al. (36) signifying amplified defects in the gene expression of virulence genes for flagellin synthesis, motility, and bile resistance in pathogenic *Salmonella* strain 14028 (34). To date, studies centered on the modulation of responses in the host immune system to attenuated *Salmonella* Dam-serovars have indicated that greater immunity is conferred to vaccinated hosts (36) and may be highly effective as live vaccines against murine typhoid fever in an increased attenuated state (33, 45) taking in consideration the persistence of Dam mutants in infected animals (46).

In contrast, Dam^−^ mutants of *E. coli* have demonstrated increased expression for numerous genes including s*ul*A, *trp*S, *trp*R, *tyr*R, and *gln*S, within which GATC sites are localized within the −10 and −35 region of their promoters (34, 47). Likewise, the increased adherence and actin pedestal formation on cultured mammalian cell lines for Δ*dam* mutants of Enterohemorrhagic *E. coli* (EHEC) OH157:O7 when compared to wild type strains speaks to the epigenetic effect of *dam* on various bacterial species (26). Within this milieu, this study was conducted to determine the epigenetic influence of *dam* on growth, fluoroquinolone resistance, gene expression, and human uroepithelial cell attachment in UPEC by the employment of lambda (λ) red recombineering.

## Materials and Methods

### Bacterial Strains, Growth Conditions, and Culture Medium

A total of 174 non-duplicate uropathogenic fluoroquinolone-resistant *E. coli* strains isolated from patients diagnosed with uncomplicated UTI were analyzed prior to epigenetic studies (48, 49). Uropathogenic control strain *E. coli* CFT073 [genotype *amp*^R−^, *dam*^+^, MDR^−^, *qnr*A^−^, QRDR^−^, *pap*EF^+^] (50) and fluoroquinolone-resistant *qnr*A^−^, and cured quinolone-resistance determining region (QRDR)-positive *E. coli* clinical isolates A620b, C119, U155 [genotype *amp*^R−^, *dam*^+^, MDR^+^, *qnr*A^−^, QRDR^+^, *pap*EF^+^] (48) were utilized for epigenetic studies. Fluoroquinolone-resistant *qnr*-positive control strains *E. coli* strain Lo QnrA^+^ and *E. coli* J53 pMG252 were generously donated by Dr. G. A. Jacoby and Prof. P. Nordmann, respectively. *E. coli* ATCC 25922 and MG1655, non-pathogenic negative controls used were generously provided by the Microbiology Department at the University Hospital of the West Indies (UHWI), Mona, Jamaica and the University of Minnesota, respectively. Multidrug-susceptible uropathogenic *E. coli* CFT073 [WAM2267] and J53 Az^R^ served as control strains in antibiotic susceptibility and Dam methylation studies (ATCC, VA, USA). In addition, the laboratory *E. coli* strain MG1655 and JM109 [genotype K-12sp *rec*A^−^, *end*A^−^, F′] (Promega, WI, USA) were utilized as *dam-*positive control and competency for cloning, respectively. All isolates were routinely cultured in either Luria-Bertani (LB) medium (Difco™, BD Diagnostics, MD, USA) or Tryptic Soy agar (EMD Millipore, Merck, Darmstadt, Germany) supplemented with antibiotics including ampicillin (100 μg/ml), carbenicillin (125 μg/ml), chloramphenicol (10 or 15 μg/ml), ciprofloxacin (1 μg/ml), and nalidixic acid (40 μg/ml) (Cellgro^®^, Mediatech Inc., VA, USA or Sigma-Aldrich, MO, USA), where appropriate. When performing genetic transformations, the Super Optimal Broth (SOB) medium (AMRESCO, OH, USA) served as a nutrient-rich medium for the resuscitation of cells. All strains were incubated at 37°C unless indicated otherwise.

### Plasmids

Plasmids utilized were: (a) temperature-sensitive helper plasmid pKM208 (8731 bp) that harbors λ red genes including *bet, gam* and *exo* and *amp*^R^ gene under the control of P_tac_ promoter and *lacI* repressor (51); (b) pKD3 (2804 bp) (Genbank AY048742) to obtain the chloramphenicol acetyl transferase gene template required for linear DNA preparation for recombineering experiments (52); (c) cloning plasmid pGEM^®^-T Easy Vector (3015 bp) that encodes the *amp*^R^ gene (Promega, WI, USA) or pCR^®^II-TOPO (4.0 kb) and *amp*^R^ + *kan*^R^ conferring ampicillin and kanamycin resistance (Invitrogen, CA, USA), respectively.

### DNA Manipulations

#### *Dam* Screening

Prior to determining the putative influence of the *dam* gene on the P fimbriae attachment and quinolone resistance among fluoroquinolone-susceptible and -resistant UPEC, strains were subjected to screening for *dam*. UPEC isolates were subjected to genomic DNA extraction using the Promega Wizard Genomic DNA Extraction kit (Promega, WI, USA) according to the manufacturer’s instructions. DNA extracts were quantified using the Thermoscientific Nanodrop 2000 Spectrophotometer (Wilmington, DE, USA) followed by *dam* amplification by PCR using Promega GoTaq Green 2× kit (Promega, WI, USA), 1 μl DNA and the UR427/UR428 primer pair (Table 1) to produce an amplicon size of 1071 bp. Forward primer UR427 was located upstream the *dam* gene while UR428 was located further downstream. As a foundation for primer design, *E. coli* laboratory strain K-12 substrain MG1655 (53) was used to augment the 837 bp *dam* gene and flanking regions. PCR reactions were performed using the GeneAmp 9700 Thermal Cycler (Applied Biosystems, USA) according to the following parameters: 95°C for 5 min (initial denaturation), 29 cycles of 95°C for 30 s, 55°C for 30 s, 72°C for 1 min, and 72°C for 10 min (final extension). Amplicons were detected by UV fluorescence following electrophoresis in ethidium bromide-stained agarose gels.

**Table 1:** **Primers, sequence, and amplicons size used in epigenetic influence of Dam studies**.

Gene/region	Primer	Sequence (5′-3′)	Band Size (bp)	Reference
Dam	UR427	CTAGTCTAGATGTACGCTTCGAAAGAAGAGG	1071	This study
	UR428	CCCGCTCGAGATCAGCCGACAGAATTGAGG		
*dam* deletion (*cam*^R^ cassette)	UR429C	CACAGCCGGAGAAGGTGTAATTAGTTAGTCAGCATGAAGAAAAATCGCGTGTAGGCTGGAGCTGCTTC	1323	This study
	UR430C	TTTCATCCGCTTCTCCTTGAGAATTATTTTTTCGCGGGTGAAACGACTCCCATATGAATATCCTCCTTA		
QRDR (Topoisomerase II)	GyrA6	CGACCTTGCGAGAGAAAT	626	(54)
	gyrA631	GTTCCATCAGCCCTTCAA		
QRDR (Toposiomerase IV)	ParCF43	AGCGCCTTGCGTACATGAAT	849	(55)
	ParCF981	GTGGTAGCGAAGAGGTGGTT		
*dam* (*E. coli* CFT073)	damCFT073-F	ACTTCCATGGGACAGAATTGAGGGGGCA	996	This study
	damCFT073-R	AAGCGTCGACATCAAGGTTATCTCCCGCAA		
*qnr*A gene (pMG252)	damqnrA-F	TCTTAGTCGACAAGATCCGAAGGTCATTGAGC	1236	This study
	damqnrA-R	TCGGCCATGGATGAAGCAACCAGGCAATG		
Insert coding sequence	M13-F	CGCCAGGGTTTTCCCAGTCACGAC		
	M13-R	TCACACAGGAAACAGCTATGAC		
*pap* promoter region	papIB–F	TTTCTGAACAGGCATGATGG	418	This study
	papIB-R	GTGAGCGCTGAACCATACCT		
P fimbriae assembly	papEF-F	GCAACAGCAACGCTGGTTGCATCAT	336	(56)
	papEF-R	AGAGAGAGCCACTCTTATACGGACA		

*UR427 – XbaI restriction enzyme site*.

*UR428 – XhoI restriction enzyme site*.

#### DNA Methylation Assay

Chromosomal and plasmid DNA from *dam*-positive *E. coli* isolates A620b, cured C119 (cC119), U155, uropathogenic *E. coli* CFT073 and *dam*-positive control strain *E. coli* MG1655 were subjected to differential digestion for *dam* function with restriction endonucleases *Sau*3AI, *Mbo*I, and *Dpn*I according to Chen et al. (61) with modifications. Essentially, 0.5 μg of chromosomal and plasmid DNA was digested for 1.5 h at 37°C with 2 U *Sau*3AI (Promega, WI, USA), 10 U *Dpn*I (New England Biolabs, MA, USA), or 2.5 U *Mbo*I. *Sau*3AI cleaves DNA at GATC sites regardless of methylation state, *Dpn*I cleaves GATC sites that have a methylated adenine residue, and *Mbo*I cleaves unmethylated GATC sites. The resulting DNA was visualized under UV on ethidium bromide-stained agarose gels.

#### Dam Mutant Construction

**Figure 1:**
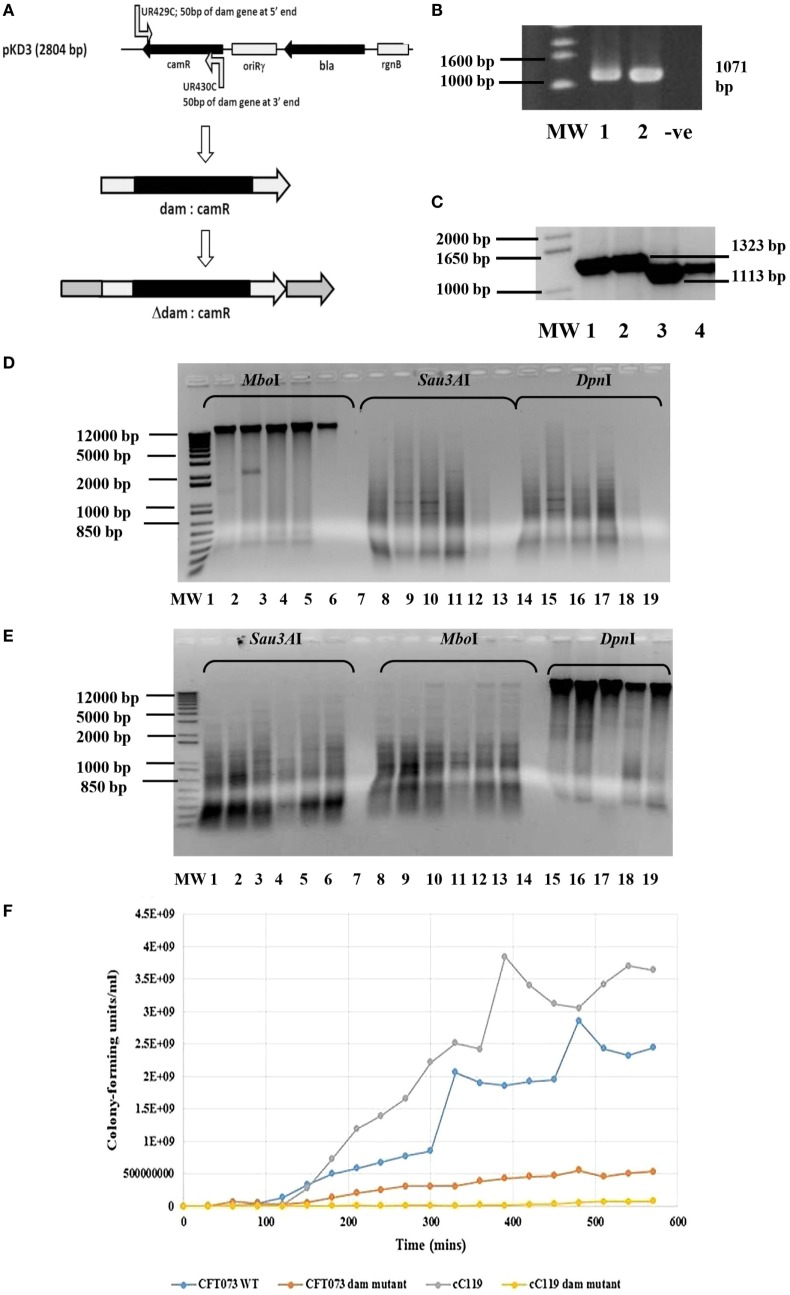
**Genotypic and growth characteristics displayed by parental and *dam-*mutant strains of UPEC**. **(A)** Schematic diagram of gene disruption strategy for chromosomal insertion of chloramphenicol resistance gene from pKD3 into *dam* gene within UPEC chromosome subsequent to λ red recombineering with pKM208. **(B)** Amplified *dam* fragment from wild type UPEC strains CFT073 (lane 1) and cured parental strains C119 (lane 2) to produce 1071 bp amplicon. MW is 1 kb DNA ladder (Bioneer Corporation, Republic of Korea) and −ve is negative control. **(C)** PCR screening of UPEC candidates for *dam* mutation observed as 1323 bp products using primers UR427 and UR428. MW is a 1 kb Plus DNA ladder (Invitrogen, USA). **(D)** Dam methylation pattern in UPEC CFT073 wild type (lanes 1, 2, 8, 9, 14, 15), C119 wild type (lanes 3, 4, 10, 11, 16, 17), and *E. coli* K-12 substrain MG1655 (5, 12, 18) strains subsequent to digestion with *Mbo*I, *Sau*3AI, and *Dpn*I. The negative control (7, 13, 19) and 1 kb Plus DNA ladder (MW) are also shown. **(E)** Dam methylation pattern in UPEC *dam* mutants CFT073 (lanes 1, 2, 3, 8, 9, 10, 15, 16, 17) and C119 wild-type (lanes 4, 5, 6, 11, 12, 13, 18, 19) subsequent to digestion with *Sau*3AI, *Mbo*I, and *Dpn*I. The negative control (lanes 7, 14) and 1 kb Plus DNA ladder (MW) are also shown. **(F)** Growth curve (CFU/milliliter versus time) for UPEC strains CFT073, CFT073 Δ*dam*, cC119, and cC119 Δ*dam*.

*dam* deletion mutants were created *via* the λ red recombinase system involving a modified one-step allelic exchange and inactivation protocol as proposed by Datsenko and Wanner (52) and illustrated in Figure 1A. Clinical and control UPEC strains containing the Red recombinase helper plasmid pKM208 were generated following electroporation using the BIORAD Gene Pulser X-cell Electroporation System (2 kV, 25 μFD, and 200 Ω). After recovery of cells in SOC medium (Invitrogen, CA, USA) at 37°C for 1 h, 100 μl of the electroporation mixture was plated in triplicate on LB agar with 125 μg/ml carbenicillin and incubated overnight with agitation at 30°C to maintain the replication of the temperature-sensitive pKM208 within the cells.

To construct the DNA fragment to be used for allelic exchange to make the *dam* deletion mutation, PCR amplification of the chloramphenicol cassette (*cam*^R^) was conducted using the pKD3 template (52) and the *dam* insertion primer pair UR429C/UR430C. Each primer carried extra sequence overhangs of 50 bp genome homology to either the 5′ end (UR429C) or 3′ end (UR430C) of the *dam* gene, followed by approximately 20 bp sequence homology to the chloramphenicol resistance gene (*cam*^R^) to generate an amplicon that inserted the resistance gene roughly 50 bp into the coding sequence of the *dam* gene (Δ*dam*:*cam*^R^ fragment). PCR parameters included initial denaturation at 95°C for 5 min, 29 cycles of 95°C for 30 s, 55°C for 30 s and 72°C for 1 min 30 s, and final extension at 72°C for 10 min. Following electrophoresis, the 1113 bp band was purified by gel extraction by the QIAquick Gel Extraction kit (QIAGEN Sciences Inc., MD, USA) according to the manufacturer’s instructions in a final volume of 50 μl water and quantitated as previously described.

Electrocompetent suspensions of IPTG-induced (1 mM) *E. coli* strains A620b, cC119, U155, and CFT073 (all harboring pKM208) were electroporated with 5 μl of purified Δ*dam*:*cam*^R^ PCR product and recovered in SOC medium. Recombinant clones were selected on LB agar supplemented with 10 μg/ml chloramphenicol and incubated at 37°C. To facilitate the loss of pKM208 from carbenicillin-resistant transformants, previously screened transformants were plated on medium with 10 μg/ml chloramphenicol and incubated at 42°C overnight. Recombinant clones were verified by plating on medium containing the appropriate antibiotic for incubation at 37°C. Selected strains were chloramphenicol resistant and carbenicillin sensitive. Presumptive colonies were further confirmed for the presence of the Δ*dam*:*cam*^R^ allele (1323 bp) by PCR using primers UR427/UR428 as previously described prior to *dam* methylation assays and observations under the light microscope (WARDS Natural Science Establishment, Inc., NY, USA).

#### Complementation of *Dam* and *dam*qnrA

Complementation vectors pGEM*dam* or pGEMQA were constructed by inserting the *dam* and *qnr*A genes amplified from *E. coli* CFT073 and C119 wild-type, respectively, into Promega pGEM^®^-T Easy vector system (Promega, WI, USA) according to the manufacturer’s recommendations. PCR amplification was conducted individually using the corresponding laboratory-designed primer pairs damCFT073-F/damCFT073-R and damqnrA-F/damqnrA-R (Table 1) under parameters suitable for selected sequence. The DNA band of interest was excised and gel purified (IBI Scientific, KappCourt, IA, USA) followed by cloning in pGEMdam or pGEMQA according to the Promega pGEM^®^-T Easy vector system. Subsequently, presumptive colonies were screened by PCR, subjected to genomic and plasmid DNA extraction and quantification as previously described. Complement strains were further tested for *dam* function by the Dam methylation assay and visualized under the light microscope (WARDS Natural Science Establishment, Inc., NY, USA).

### Growth Rate Studies

To investigate the impact of *dam* on UPEC growth rate, wild-type (CFT073), cured parental (C119), Δ*dam* and complement Δ*dam* strains of *E. coli* CFT073 and C119 were grown to saturation in LB with appropriate antibiotics at 37°C, the growth rate measured by UV/Vis spectrophotometer (Cecil CE 9000 series, Cecil Instruments Limited, Cambridge, UK) and viable cell count method according to a modified protocol by Matlock et al. (62). A 2% batch culture was prepared by transferring 2 ml of an overnight culture in 100 ml. The culture was grown for up to 10 h, during which the optical density at 600 nm and associated viable cell count (CFU/milliliter) was determined following duplicate plating on LB agar incubated overnight at 37°C. Results were obtained from three separate experiments. Growth rate (*k*) was calculated as:

$$k=\frac{\log10\left[X_{t}\right]-\log10[X_{0}]}{0.301\times t}=\mathrm{gen/h}$$

where *X*_t_ is the higher CFU/milliliter, *X*_0_ is the lower CFU/milliliter, and *t* is the time interval between both (in hours).

Generation time (*t*_gen_) was calculated as:

$${\text{t}}_{\text{(gen)}}=\text{1/}k=\text{h/gen}$$

### Antimicrobial Susceptibility

Wild-type and Δ*dam-*mutant strains of *E. coli* CFT073 and cured C119 as well as control strains *E. coli* strain Lo QnrA^+^ and *E. coli* JM109 harboring pGEMQA were subjected to antimicrobial susceptibility testing using Sensititre Substrate-in-Well GNUR2F Gram-negative MIC plates (TREK Diagnostic Systems, Inc., OH, USA) for inoculation and incubation (63). A 50 μl suspension of the sample was used to inoculate Sensititre plates, sealed and incubated at 35°C for 18–24 h. The plates were observed for the presence of a growth button at the base of the microtiter well and fluorescence intensity (+++ to 0) captured with a UV Benchtop Variable Transilluminator and Photo Doc-It Imaging System (UVP, CA, USA).

### Biofilm Analysis

The correlation of *dam* with oxygen and antiobiotic pressure on the formation of biofilms in the presence and absence of 2% d-mannose (AMRESCO, OH, USA) were explored. First, overnight cultures of bacterial strains were diluted 1:10 into Mueller-Hinton broth, 0.2 ml transferred into 96-well polystyrene microtiter plates (Nunc-Immuno Microwell) and incubated at 37°C for 24 h without agitation. Oxidative stress studies were initiated by the addition of hydrogen peroxide (H_2_O_2_) to microwell cultures to a final concentration of 0, 0.1, 0.2, 0.3, 1, or 2 mM, prior to incubation and processing as indicated by a modification to concentrations utilized by Hedge et al. (64) and Hryckowian et al. (65). Likewise, for the effect of the antimicrobial agents, the quinolones, nalidixic acid (0, 8, 24, 32, 40, or 48 μg/ml), and ciprofloxacin (0, 1, 2, 3, 4, or 5 μg/ml) were added to microwell cultures prior to incubation. Post 24 h incubation, wells were decanted, washed thrice with 1× phosphate-buffered saline (PBS, pH 7.4), and adhered bacterial cells stained with 200 μl 0.1% crystal violet for 15 min at room temperature. Unbound dye was decanted, wells washed thrice, and dried. Stained cells were dissolved with 95% ethanol for 15 min, and optical density measured at 570 nm using Victor X Multilabel Plate Reader (PerkinElmer, MA, USA). All experiments were conducted in triplicate from three independent experiments (20). Statistical data analysis was conducted using the Student’s *t* test for variance in data collected from test strains when compared to parental or wild type (SPSS software, Version 20, SPSS Inc., USA). A *p* value of <0.05 was considered statistically significant.

### Transcriptional Analysis

In order to determine the impact of *dam* on DNA transcription, cured parental, *dam* mutant and complement strains of C119 and CFT073 were cultured overnight then subjected to RNA isolation and purification using the Promega SV RNA Isolation Kit (Promega, WI, USA) according to the manufacturer’s instructions. Reverse Transcription was conducted using the GoScript™ Reverse Transcriptase System First-Strand Synthesis System (Promega, WI, USA) to synthesize first-strand cDNA as instructed by the manufacturer. cDNA was amplified by means of semi-quantitative PCR (sq-PCR) for *dam, pap*IB, *pap*EF, *qnr*A, and the housekeeping genes (*arc*A, *gyr*B, *mdh, rec*A, and *rpo*S) expression in *E. coli* C119 and CFT073 parental, mutant, and complement strains using GoTaq^®^ Green Reaction Mix and gene-specific primers (Tables 1 and 2), annealed at 55°C for 1 min. Ten microliter samples were taken at cycles 23, 25, 30, and electrophoresed in ethidium bromide-stained agarose gels (66).

**Table 2:** **Primers, sequence, and amplicons size used in epigenetic influence of Dam studies (continued)**.

Gene/region	Primer	Sequence (5′–3′)	Amplicon size (bp)	Reference
Aerobic respiration control protein	arcA-F	GAAGACGAGTTGGTAACACG	645	(57)
	arcA-R	CTTCCAGATCACCGCAGAAGC		
DNA gyrase subunit B	gyrB-F	TCGGCGACACGGATGACGGC	911	(58)
	gyrB-R	ATCAGGCCTTCACGCGCATC		
Malate dehydrogenase	mdh-F	ATGAAAGTCGCAGTCCTCGGCGCTGCTGGCGG	932	(58)
	mdh-R	TTAACGAACTCCTGCCCCAGAGCGATATCTTTCTT		
ATP/GTP-binding motif	recA-F	CGCATTCGCTTTACCCTGACC	780	(58)
	recA-R	TCGTCGAAATCTACGGACCGGA		
Transcription factor sigma S	rpoS-F	TATGAGTCAGAATACGCTGAAA	Varies	(59)
	rpoS-R	GGAACAGCGCTTCGATATTCAG		
Quinolone-resistance gene	qnrA-F	ATTTCTCACGCCAGGATTTG	516	(60)
	qnrA-R	GATCGGCAAAGGTTAGGTCA		

### Phenotypic Influence of *Dam* Methylation on P Fimbriae

The *pap*I-B pili regulatory region was identified using the *pap*IP1for/*pap*IP1rev primers as described by Holden et al. (67). The concentrated, purified 418-bp PCR product of the *pap*I-B regulatory region from strains U155 and C119 (~1.8 ng in 3.5 μl) was cloned into Invitrogen pCRII^®^ TOPO vector (Invitrogen, CA, USA) according the manufacturer’s instructions to construct pSAMS1 and pSAMS2, respectively, further used to transform One Shot *E. coli* competent cells (Invitrogen, CA, USA). Transformants were selected on LB supplemented with 50 μg/ml carbenicillin/40 mg/ml X-gal/100 mM IPTG, prior to recombinant vector purification by QIAprep Spin Miniprep kit (QIAGEN Inc., CA, USA). The cloned *pap* intergenic DNA insert was confirmed by colony PCR with M13F/M13R primers, sequenced at the Virginia Commonwealth University Core Facility in Richmond, VA, USA and analyzed using NCBI BLAST. *dam* methylation of the *pap*I–*pap*B insert was determined as previously described in the Materials and Methods prior to pSAMS digestion by *Eco*RI (Promega, WI, USA) to release the intergenic region.

### *pap*I Expression Assay

In order to determine variation in *pap*I expression, wild-type and *dam-*mutant strains of cC119 and CFT073 were cultured overnight then subjected to RNA isolation and RT-PCR using the QIAGEN RNeasy Mini kit, Invitrogen Superscript™ First-Strand Synthesis System (Invitrogen, CA, USA), respectively according to the manufacturer’s instructions. cDNA templates were amplified by means of semi-quantitative PCR using *pap*ICFT073-1F/*pap*ICFT073-1R primers (Table 1) as described by Holden et al. (67). Ten microliter aliquots were processed as previously noted.

### Attachment Assays

Urinary human cell lines, HEK-293 (human embryonic kidney cells) and HTB-9 (human bladder cells) (ATCC, VA, USA) were individually cultured in 25-cm^2^ flasks prior to washing attached cells with 10 ml Dulbecco’s phosphate-buffered saline (D-PBS) before trypsin (ATCC) exposure. Cells were incubated for 10 min at room temperature until the monolayer was almost detached, followed by the addition of 9 ml minimal essential media (MEM) with 10% fetal calf serum (ATCC) and amino acids (Cellgro^®^), and RPMI-1640 media with glucose, HEPES and 10% fetal calf serum for HEK-293 cells and HTB-9 cells, respectively. Cells and suitable medium (1:1) were added in duplicates to six-well cell culture plates following 1:4 areal dilution and incubated at 37°C in 5% CO_2_ for 48 h until confluent. One set of cell culture plates was utilized for assessment of total *E. coli* cells present, and the other for assessment of adherent *E. coli* cells only. Simultaneously, *E. coli* control, cured parental, and *dam-*mutant strains of C119 and CFT073 were cultured in LB without shaking for 48 h. Prior to the attachment assay, MEM (HEK-293 cells) and RPMI-1640 (HTB-9 cells) were aspirated from the wells and 1 ml of the appropriate medium supplemented with 20% (1.1M) d-mannose (Sigma-Aldrich) added to each well. Furthermore, 10 μl bacterial culture (OD = 1 at 600 nm) mixed with 1.8 μl of 20% d-mannose was added to each well. Samples were evenly distributed, centrifuged for 5 min at 600 × *g*, and incubated at 37°C in 5% CO_2_ for 1.5 h. At the end of incubation, plates designated for the detection of adherent *E. coli* cells were washed five times with Dulbecco’s PBS with 1 mM CaCl_2_ and 0.5 mM MgCl_2_ (PBS^2+^), 40 μl Triton X-100 added to lyse the cells, and the bottom of wells scraped. Sterile PBS^2+^ was added to the wells and mixed before serially diluting to 10^4^ (100 μl sample to 900 μl PBS^2+^) for plating on LB agar. In contrast, plates selected for the determination of total number of bacteria were not subjected to washing but direct treatment with Triton-X, scraping, serial dilution and plating on LB agar. All plates were incubated overnight at 37°C, and CFU/milliliter determined and analyzed.

## Results

### Dam Methylation Pattern, Growth Rate and Phenotypic Characteristics of UPEC *Dam* Mutants

Of the 174 FQ-R resistant isolates including cured UPEC strains A620b, C119, U155, CFT073, and MG1655 screened, 71% were positive for the *dam* sequence amplified by UR427/UR428 to produce a 1071 bp product (Figure 1B). Chromosomal digestion of parental UPEC strains cC119 and CFT073 with methylation-sensitive enzymes (*Dpn*I and *Mbo*I) revealed the presence of functional *dam* capable of GATC methylation within wild-type strains (Figure 1D). To assess the putative role of *dam* on virulence, *dam*-deficient mutant strains were successfully constructed using Red-recombinase-mediated allelic exchange as the wild-type *dam* gene was interrupted with a chloramphenicol-resistance (*cam*^R^) gene (Figure 1A). Two UPEC strains (C119 and CFT073) were successfully transformed with pKM208. Successful replacement of the wildtype *dam* gene was selected for by growth of chloramphenicol-resistant Δ*dam* colonies and PCR amplification of a 1323 bp product due to the Δ*dam*:*cam*^R^ allele (Figure 1C). Further confirmation was revealed by a total loss of DNA adenine methylation and methylase-sensitive digestion among *dam* mutants (Figure 1E).

**Figure 2:**
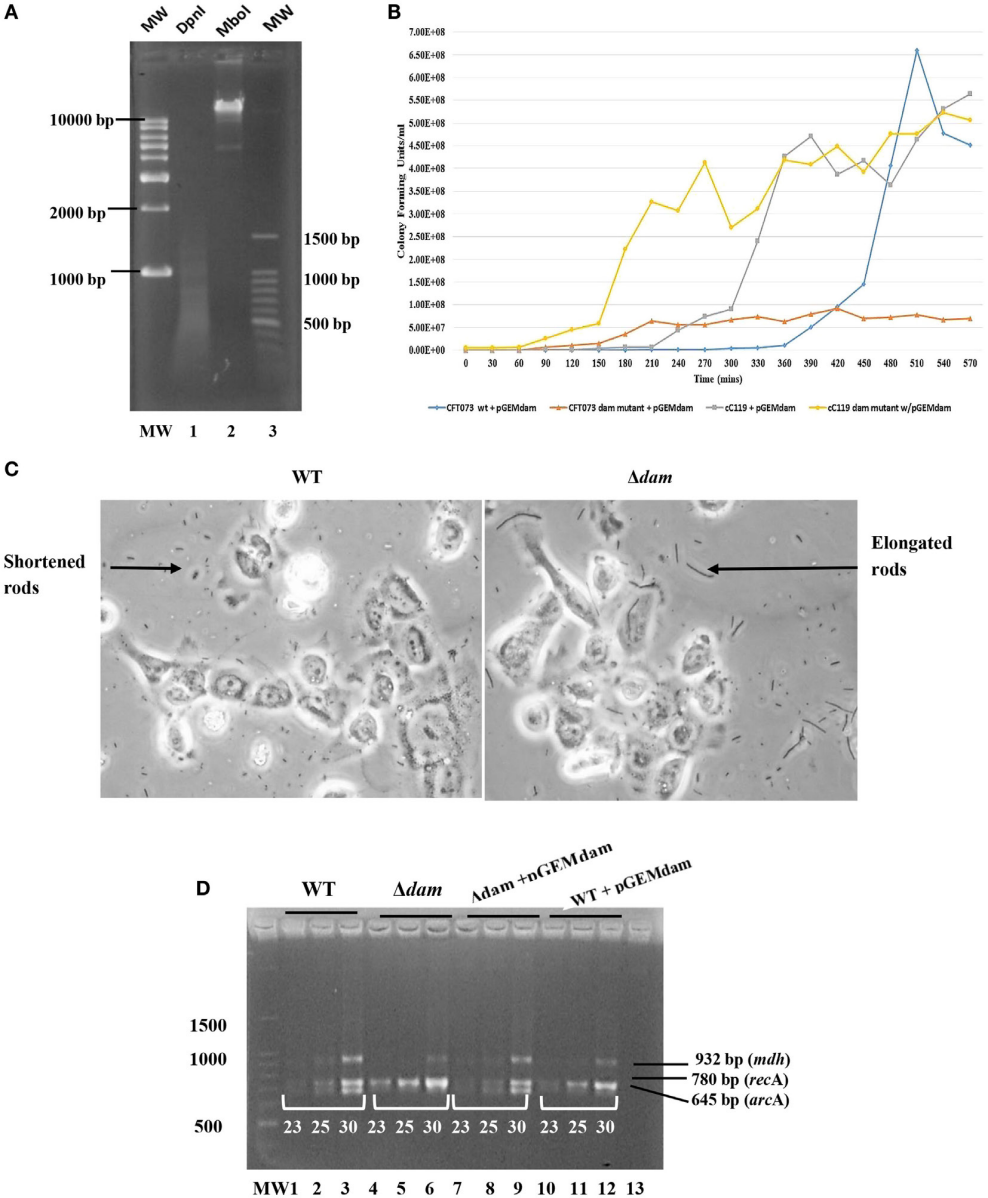
**Genetic and growth characteristics displayed by *dam-*complemented mutant strains of UPEC relative to wild-type**. **(A)** Dam methylation pattern in UPEC CFT073 strain subsequent to digestion with *Dpn*I (lane 1) and *Mbo*I (lane 2). The 1 kb plus DNA ladder (MW) is also shown. **(B)** Growth curve (CFU/milliliter versus time) for *dam* complement UPEC strains of CFT073, CFT073 Δ*dam*, cC119, and cC119 Δ*dam*. **(C)** Micrographs for wild-type (WT) and *dam* mutant (Δ*dam*) UPEC strains, illustrating the morphological occurrence of shortened- and filamentous rods, respectively. **(D)** Semi-quantitative RT-PCR for *mdh, rec*A, and *arc*A expression at cycles 23, 25, and 30 for CFT073 (lanes 1–3), CFT073 Δ*dam* (lanes 4–6), CFT073 + pGEMdam (lanes 7–9), CFT073 Δ*dam* + pGEMdam (lanes 10–12). The 100 bp molecular marker MW (Promega, WI, USA) and negative control are shown (lane 13).

Microscopic observations revealed phenotypic variation in the population of *dam-*mutant strains: elongated bacilli or interconnected filamentous cells contributed up to 60% of the total population when compared to the shortened rods exclusively exhibited by wild-type strains (Figure 2C). Given that average length of wild type *E. coli* cells can vary between 1.6 and 3.9 ± 0.9 μm according to growth phase and condition (68), cell measurements were largely excluded. Despite this, filamentous cells demonstrated an estimated increase in cell length by up to eight times that of wild-type strains.

### Complementation of *Dam* Mutants

To successfully construct the complementation plasmid pGEM*dam* or *qnr*A-containing pGEMQA, the corresponding *dam* (996 bp) or *qnr*A (1236 bp) amplicon from wild-type strains were used as inserts for cloning into the vector pGEM-T^®^ Easy. Furthermore, plasmids were also successfully transformed into study strains (cC119 and CFT073) for further analysis. Micrographs and *dam* function analysis in *dam* complement strains reflected the successful morphological and phenotypic restoration of *dam* denoted by the predominance of shortened rods and regular *dam* methylation (Figure 2A) as displayed by paternal strains.

### Growth Rate Studies

Despite exhibiting comparable spectrophotometric growth trends to cured parental strains, Δ*dam* mutants exhibited at least twofold reduction in actual growth rate, cell numbers, and an extended lag phase (90–180 min) before initiating logarithmic growth in broth cultures (Figure 1F). There were distinguishable differences in colony forming unit per milliliter over time among all four strains titred to determine viable cell counts. Δ*dam* mutants were significantly lower (~4.5- to 50-fold) in numbers when compared to wild-type counterparts at the end of log phase. Generation time (*t*_gen_) derivations were 13.5 min/gen (CFT073 wt), 32.3 min/gen (CFT073 Δ*dam*), 28.2 min/gen (cC119), and 55.32 min/gen (cC119 Δ*dam*); an approximate 50% reduction in growth rate of *dam-*mutant strains.

On the other hand, the restoration of the *dam* function by complementation for cured parental and Δ*dam* strains, resulted in discernable decreases in viable count over time when compared to strains lacking the plasmid (Figure 2B). *dam* complement strains produced marginally higher values than wild types harboring the *dam* plasmid; the lag phase of plasmid-bearing wild types being as high as 330 min. Furthermore, respective generation time derivations of 28.8, 35.4, 56.4, and 26.4 min/gen for CFT073 + pGEM*dam*, CFT073 Δ*dam* + pGEM*dam*, cC119 + pGEM*dam*, and cC119 Δ*dam* + pGEM*dam* illustrated the role of the *dam* plasmid in suppressing cell division defects linked to Dam.

### Antimicrobial Susceptibility of pGEMQA-Positive Δ*dam* Mutants and Wild Type Strains

Susceptibility responses to various antimicrobial agents varied among wild type, Δ*dam*- and pGEMQA-harboring stains (Table 3). A decrease in resistance by eightfold was noted for cC119 versus cC119 Δ*dam* against amoxicillin/clavulanic acid, trimethoprim/sulfamethoxazole and gentamicin, and fourfold against ciprofloxacin. For CFT073 Δ*dam*, a 32-fold (each) and 64-fold increase in resistance was identified against amoxicillin/clavulanic acid, ampicillin, and carbenicillin, respectively. In contrast, the resistance response of *dam* mutant *qnr*A complement cC119 strain was similar to the parental strain, with the restoration of resistance by eightfold (trimethoprim sulfamethoxazole and gentamicin) and fourfold (amoxicillin/clavulanic acid and ampicillin). Likewise, *dam*-mutant *qnr*A complement CFT073 maintained the susceptibility pattern identified among Δ*dam* strains.

**Table 3:** **Antimicrobial susceptibility responses of *E. coli* wild type (wt), cured (c), *dam* mutant, and pGEMQA-bearing strains subjected to the sensititre substrate-in-well GNUR2F Gram-negative MIC plate test**.

Strain	MIC (μg/ml)								
	AMP	AUG2	AXO	CAR	CIP	FEP	GEN	SXT	NIT
CFT073 wt	0	0	0	0	0	0	0	0	0
CFT073 Δ*dam*	32	32/16	0	64	0	0	0	0	0
CFT073 wt + pGEMQA	32	32/16	0	64	0	0	0	0	0
CFT073Δdam + pGEMQA	32	32/16	0	64	0	0	0	0.5/9.5	0
cC119	32	32/16	0	64	4	32	8	4/76	0
cC119 Δ*dam*	8	8/4	0	32	1	0	0	0.5/9.5	0
cC119 wt + pGEMQA	32	32/16	0	64	4	4	0	4/76	0
cC119 Δ*dam* + pGEMQA	32	32/16	0	64	2	0	8	4/76	0
Lo QnrA^+^	32	8/4	0	64	1	0	8	4/76	0
JM109 pGEMQA	32	16/8	0	64	0	0	0	4/76	0

*AMP, ampicillin; AUG2 – amoxicillin/clavulanic acid; AXO, ceftriaxone; CAR, carbenicillin; CIP, ciprofloxacin; FEP, cefepime; GEN, gentamicin; SXT, trimethoprim/sulfamethoxazole; NIT, nirofurantoin*.

### Transcriptional Analysis

The twofold increased expressions of *rec*A and *pap*IB were noted among Δ*dam* strains compared to cured strains of C119 and CFT073 (Figure 2D). Remarkably, results indicated up to threefold increase in expression among Δ*dam* strains bearing pGEMQA. With the restoration of *dam* through pGEM*dam*, Δ*dam* strains reverted to the original level of expression demonstrated by wild types. Parental strains harboring pGEMdam did not display alterations in *rec*A expression. However, Δ*dam* strains harboring pGEMQA displayed elevated *qnr*A levels (twofold) relative to cured parental strain, while those with pSAMS presented up to five times elevation in *pap*IB expression relative to parental strains. The impact of *dam* deficiency on the expression of *arc*A and *mdh* was revealed by reduced expression among *dam* mutants by 0.5-fold except for strain CFT073 (3× lowered *arc*A expression). *pap*EF expression was singly identified in CFT073 + pGEMQA strains at extremely low levels. Equally, *dam-*mutant strains did not express *dam*. However, *dam* restoration was observed though 0.5 times lower in intensity relative to parental strains.

### Biofilm Analysis

**Figure 3:**
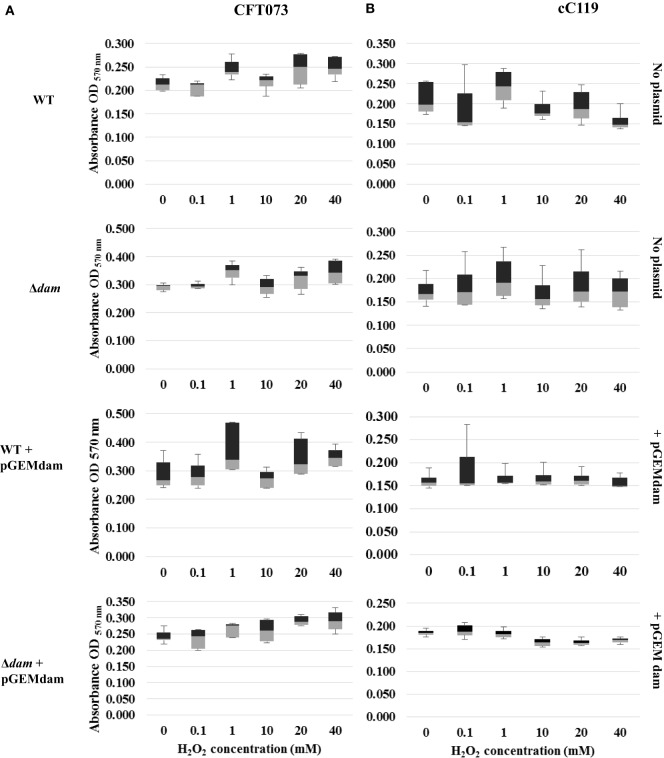
**Distribution analysis of biofilm formation for wild type (WT), Δ*dam*, WT + pGEMdam, and Δ*dam* + pGEMdam UPEC strains of CFT073 (A) and cC119 (B) in various hydrogen peroxide (H_2_O_2_) concentrations**.

**Figure 4:**
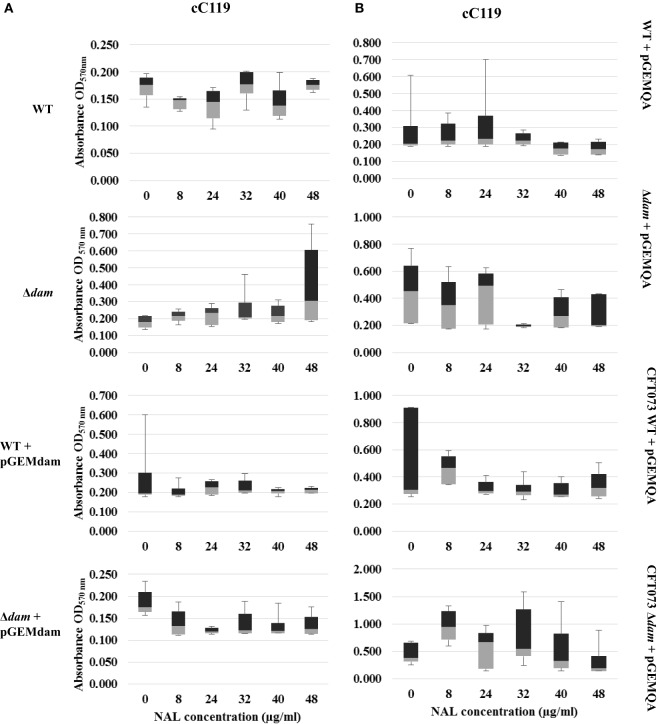
**Distribution analysis of biofilm formation for wild type (WT), Δ*dam*, WT + pGEMdam, Δ*dam* + pGEMdam, pGEMQA-harboring UPEC strains of CFT073 (A) and cC119 (B) in various peroxide and nalidixic acid (NAL) concentrations**.

The impact of oxidative stress and quinolone presence on the formation of biofilms formed by cured parental and *dam-*mutant strains (with or without complementation plasmid) was examined to simulate cell aggregation intensity during infections. Overall, our findings point to greater levels of biofilm formation based on *A*_570_ values among plasmid-free *dam* mutants than wild-type strains in the absence of environmental stress. (Figures 3 and 4). Box and whisker plots for biofilm formation in the presence of peroxide stress revealed the discernible positive impact of *dam* mutation on UPEC biofilm formation in all concentrations tested. Interestingly, all phenotypes except wild-type strains harboring pGEMdam attained elevated values at 1 mM with *dam* complemented strains, exhibiting median values analogous to both mutant and cured wild type. Exposure to increasing concentrations of peroxide resulted in elevated levels of biofilm formation (up to 1 mM for CFT073 strains), followed by a 20% gradual decrease among cC119 strains (Figures 3A,B). OD readings were notably higher for plasmid-free Δ*dam* strains when compared to cured parental strains despite lower biofilm percentages. This was denoted by the general decrease up to 40 mM notwithstanding CFT073 mutant strains’ increase in biofilm formation (*p* < 0.001). Furthermore, cell aggregation detection among CFT073 was 30% greater than cC119 strains. Dam-complemented strains of CFT073 were also shown to revert to comparable and appreciably stable levels of surface adherence as the wild-type, with increasing peroxide concentration.

In contrast, exposure to quinolones-favored biofilm-forming capabilities of mutant strains as median values increased from 0 to 8 μg/ml NAL (Figures 4A,B). Correspondingly to oxidative stress studies, wild type strains (particularly those carrying pGEMdam) exhibited poor biofilm formation as values obtained were more skewed than mutant strains. Remarkably, the pronounced effects of plasmid pGEMQA on cured parental and *dam-*mutant strains were apparent since increased levels of biofilm formation were obtained despite increasing quinolone concentration.

The biofilm formation ability of plasmid-free parental strain C119 served as the baseline for quinolone pressure comparisons to Δ*dam* strains and statistical analysis, except where CFT073 harbored pGEMQA, since wild type phenotypes of the strain are susceptible to antimicrobial agents. Supplementation with nalidixic acid resulted in an overall inhibition, and decrease in biofilm formation as NAL concentration increased (Figures 4A,B). Notwithstanding this inhibition, proliferation among *dam-*mutant strains was marginally higher in contrast to wild type, notably among CFT073 mutant strains carrying pGEMQA (twofold increase at 8 μg/ml NAL). Furthermore, NAL resistance and biofilm formation in cC119 *dam-*mutant strains harboring pGEMQA (*p* < 0.05) were restored to levels similar to the plasmid-free NAL-resistant parental strains. Studies with second generation quinolone, ciprofloxacin, were limited to cC119 for reasons indicated beforehand. Similar outcomes including the initial proliferation of plasmid-free cC119 Δ*dam* strain at 0 μg/ml (*p* < 0.05) were observed. However, steady decreases up to 5 μg/ml CIP were noted, notwithstanding proliferation at 1 μg/ml. Marginally higher levels of biofilm formation were observed among Δ*dam* strains, steadily decreasing with an increase in CIP concentrations. Notably, mutant cells harboring pGEMQA exhibited fluctuations in response in comparison to steady levels of cell aggregation displayed by parental and Δ*dam* strains harboring pGEMdam.

### Role of *Dam* in *pap*I-B Methylation

**Figure 5:**
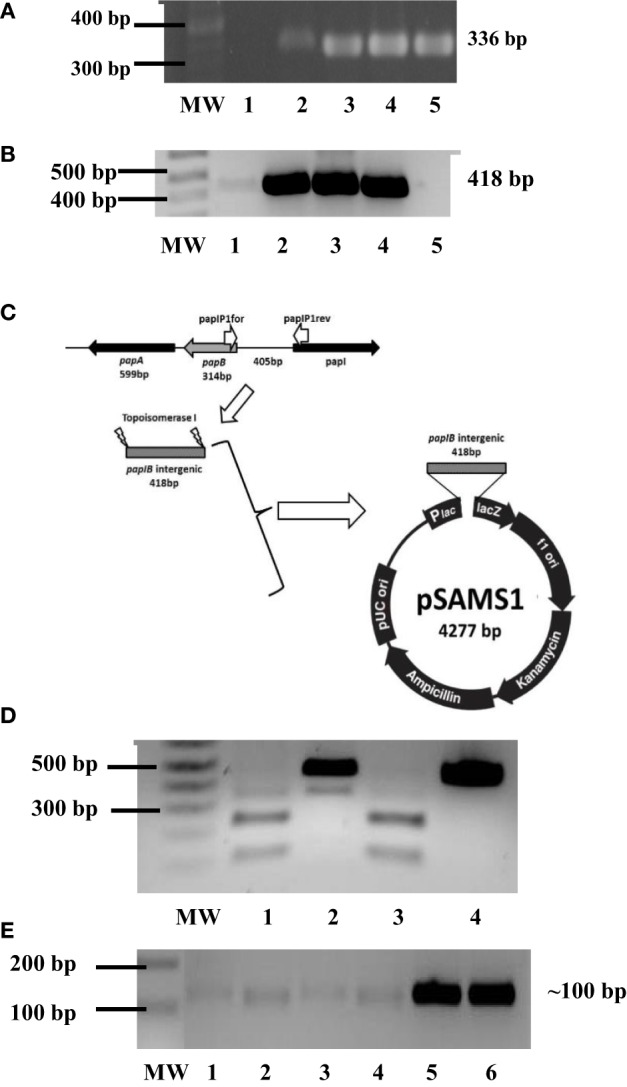
**Phenotypic influence of Dam on P fimbriae**. **(A)** PCR screening for *pap*EF in UPEC strains cC119 (lane 4), CFT073 (lane 5), and cU155 (lane 6). The 100-bp molecular weight marker (Invitrogen), negative control and positive control (*E. coli* strain Lo *qnr*A^+^/*pap*EF^+^) are represented as MW, 1 and 2, respectively. **(B)** PCR screening for *pap*I–*pap*B intergenic regulatory region in UPEC strains from UPEC strains cC119 (lane 2), CFT073 (lane 3), and cU155 (lane 4). The 1-kb plus molecular marker (Invitrogen, CA, USA), negative control, and positive control (*E. coli* strain Lo *qnr*A^+^/*pap*EF^+^) are represented as MW, 2 and 5, respectively. **(C)** Schematic representation of pSAMS1 recombinant plasmid containing cloned *pap*IB insert within pCRII–TOPOII vector. **(D)** Dam methylation patterns for *pap*I-B regulatory region. *Sau*3AI (lane 2), *Mbo*I (lane 3), and *Dpn*I (lane 4) digests of pSAMS2 isolated from cC119 are shown. MW represents the 1 kb Plus molecular marker (Invitrogen). An undigested *pap*IB fragment (lane 5) is also represented. **(E)** Semi-quantitative (sq) RT-PCR for *pap*I expression in cC119 (lane 1), cC119 Δ*dam* (lane 2), CFT073 wild-type (lane 3) and CFT073 Δ*dam* (lane 4). The 1 kb Plus molecular marker (Invitrogen) and amplified chromosomal DNA for UPEC strains cC119 and CFT073 are shown in lanes MW, 5 and 6, respectively.

*pap*I’s role in the regulation of the P fimbriae epigenetic switch also led explorations on whether *pap*I-B regions were methylated and if there were discrepancies in *pap*I expression for *dam*-deficient and *dam*-positive cells from UPEC clinical isolates. To begin examining these questions, the initial detection of P fimbriae *via pap*EF (336 bp) screening was coupled to *pap*I-*pap*B (418 bp) detection (Figures 5A,B) and cloning into pCR^®^II-TOPO10^®^ to successfully produce recombinant plasmids pSAMS1 (cU115) and pSAMS2 (cC119) (Figure 5C). For further testing on the interaction of *dam* and *pap*I-B, UPEC Δ*dam* strains and non-pathogenic control strain *E. coli* JM109 were successfully transformed with pSAMS, the *pap*I–*pap*B insert excised by *Eco*RI and subjected to a *Dam* methylation assay to reveal methylation (Figure 5D). Moreover, semi-quantitative RT-PCR used to deduce the potential influence of *dam* on the expression of *pap*-associated genes and compare *pap*I expression in cured parental and *dam* mutant UPEC strains reflected an approximate twofold increase in the transcription level of the *pap*I gene in Δ*dam* strains (Figure 5E).

### Effect of *Dam* on UPEC’s Attachment to Mammalian Cells

To examine the effect of Dam on UPEC’s interaction with mammalian cells, the ability of *dam*-deficient versus *dam*-proficient UPEC cells to adhere to cultured uroepithelial cells *in vitro* was examined. Δ*dam* strains demonstrated threefold (CFT073) to fourfold higher (cC119) cell binding levels to HEK-298 cells than their parental counterpart (Table 4). Similarly, there was threefold increase in binding to HTB-9 cells for CFT073 Δ*dam* mutants. We also noted favorable attachment of wild-type and Δ*dam* strains to HTB-9 cells (relative to HEK-293) ranging from 16- to 6-fold (for cC119) to 9- to 7-fold (for CFT073), respectively.

**Table 4:** **Data representing mean ± SD of separate experiments for attachment assay of CFT073 wt, CFT073 Δ*dam* mutant, cC119, and cC119 Δ*dam* mutant to HEK-293 kidney and HTB-9 bladder cells**.

*E. coli* strain	Colony count					
	HEK-293			HTB-9		
	
	Mean CFU/ml (×10^4^)	Mean CFU/ml (×10^5^)		Mean CFU/ml (×10^5^)	Mean CFU/ml (×10^6^)	
	
	Adherent	Total	% Adherent	Adherent	Total	% Adherent
CFT073 wt	1.55 ± 0.35	3.02 ± 2.51	5.1	3.02 ± 2.51	11.6 ± 6.58	2.6
CFT073 Δ*dam*	0.55 ± 0.05	3.04 ± 2.91	1.8	3.04 ± 2.91	3.97 ± 0.73	7.7
cC119	5.20 ± 0.10	11.9 ± 7.73	4.4	11.9 ± 7.73	52.2 ± 18.27	2.3
cC119 Δ*dam*	1.70 ± 0	4.10 ± 2.95	4.1	4.10 ± 2.95	15.3 ± 10.01	2.7

## Discussion

The molecular basis of epidemiological studies serves as the most substantial tool in understanding the pathogenesis and transmission of pathogens. In this study, the *dam* was identified as the prime source of DNA methylation in UPEC subsequent to *dam* gene interruption. Successful disruption by allelic exchange results in the partial substitution of archetypical alleles with inserts on the bacterial chromosome. Moreover, attempts at *dam* complementation proved successful in restoration of *dam* function (suppression of elongated cells and cell division defect), growth rate, biofilm formation, and gene expression studies. The pleomorphic appearance of filamentous, elongated rods among Δ*dam* strains when compared to those of wild type origin (shortened rods) denoted phenotypic similarity among the Enterobacteriales as reported by previous studies of *E. coli* K12 (29) and EHEC OH157:O7 (26). This modification points to the impact of *dam* methylation on several cellular processes in related bacteria in the orders Enterobacteriales since Dam is essential to DNA replication, gene expression regulation and mismatch repair induced by the SOS regulon (69).

The SOS regulon constitutes a collective series of over 30 genes executing a myriad of biological effects following interference in cell division, DNA replication, or damage (70, 71). These genes (*rec*A, *lex*A, *sul*A, *pol*B, *uvr*AB, *din*DF, etc.) dispersed around the chromosome are negatively regulated and repressed (72). Cell rescue subsequent to stress exposure by exogenous DNA damaging agents (e.g., UV irradiation, nalidixic acid, superoxides, 2-aminopurine) chiefly involves the interplay of RecA and LexA proteins required in expression of the regulon (72, 73). The single-stranded DNA assemblage of coprotease RecA mediates self-cleavage of LexA, the transcriptional repressor protein to activate the transcription of SOS response genes (74). Studies on the interplay of Dam with SOS regulation confirm its role as part of the recombinational repair system since deficiency results in pleiotropic changes as seen in Δ*dam* cells (75, 76). Hence, the filamentous appearance of mutant cells are assumed to be an indirect outcome of *dam* deficiency, of which the SulA protein activated in the latter part of SOS induction inhibited cell division and extended the period for DNA repair (74). More specifically, the inhibition of FtsZ polymerization by SulA prevents septation of cells and mutant DNA transfer to daughter cells since the absence of hemimethylated DNA at the replication fork profoundly alters initiation of chromosome replication. Post-DNA repair, SOS regulation is repressed by LexA and SulA is degraded to restore normal cell division (71, 77). Filamentation in response to SOS induction by DNA damaging elements of exogenous or innate immune response (polymorphonuclear leukocytes) origins may therefore serve as a means of pathogenicity as seen in the latter stage UPEC biofilm formation than singly a mechanism of survival (71).

In regards to growth rate of *dam* mutants, the prolonged lag phase preceding the comparable logarithmic phase trend to that of parental strains was noted and has also been described in *dam* mutants of *Acinetobacter actinomycetemcomitans* (78). The reduction in cell count among mutants points to the role of Dam methylase in initiating and coordinating chromosome DNA replication, nucleoid segregation, gene expression, and ultimately cell division. In *E. coli*, the replication of *dna*A-dependent chromosome from the origin of replication (*ori*C) is minimal to lacking in the absence of complete methylation of *ori*C GATC sites by Dam (79, 80). Hemimethylated origin will remain inactive, and further prevent methylation of daughter DNA strands during DNA replication since the origin is sequestered by SeqA. SeqA sequestering represses the initiation of new DNA replication cycles, *dam* regulation of the initiator DnaA promoter synthesis similarly to OriC and eventually the cell cycle, as reinitiation of chromosome replication within the same cell cycle ensues. Subsequently, DNA replication initiation is uncoordinated (81, 82) with defects in nucleoid structure due to the inability to form compacted chromosome structure for the organization of nascent nucleoids and decatenation of topoisomerase IV (79, 82). Comparatively, the observation of typical logarithmic growth among Δ*dam* mutants to that of the wild type, confirms non-essential role of *dam* expression for viability of UPEC. However, *dam* does trigger hypervariability mechanisms, which may also impact the activation of physiological pathways required for growth under specific conditions. This is in agreement with observations made for Δ*dam* mutants of *E. coli* and *Salmonella* (30).

The extended lag phase among *dam* mutants *in vitro* may occur during acclimatization of Δ*dam* strains to the growth environment and is speculated to be a direct result of the absence of Dam’s correlative activity in initiating synchronized chromosomal replication at *ori*C and the replication fork during cell growth (83). Additionally, among *dam* plasmid-bearing wild types, prolonged lag phases may be accountable to multicopied *dam* complement plasmid strains serving as *seq*A phenocopies despite Dam overproduction resulting in the subsequent inhibition of SeqA (84). Rasmussen et al. (85) have linked the level of Dam methylase production to growth rate. Hence, the observed delay in logarithmic growth may be accountable to the lack of GATC methylation for post-replicative mismatch repair of newly replicated DNA (80) alongside SulA activation during the SOS response (74).

In the absence of Dam methylation, post-replicative errors or chronic DNA damage may trigger homologous recombination, an event heavily relied upon by Δ*dam* strains for repair subsequent to lesions, nicks or double-stranded breaks in DNA (29, 76, 80). As reported among *Salmonella* exposed to stress induced environments, such contributory factors result in increased mutability with the upregulation of the SOS regulon (86, 87). Equally, *dam* mutant UPEC strains may display increased spontaneous mutation frequency and hyperrecombinogenicity (76), the latter required to repair replication forks subsequent to numerous nicks in the single strand of DNA of the growing bacterial cell (88). The hypersensitivity and hypermutability demonstrated by *dam* mutants to agents (2-amino purine, ultraviolet light and reactive oxygen species) causing DNA mismatch, lesions or mutations have also been associated with the endonuclease activity of the MutHLS complex (79). Reports by Chen et al. (61) and Zaleski and Piekarowicz (89) similarly indicated the increased hypersensitive nature of *dam*-deficient strains to DNA-damaging agents or reactive oxygen species. Furthermore, research has shown that the Agn43 outer membrane protein involved in biofilm formation in *E. coli* is epigenetically regulated by competitive binding between OxyR (global oxidative stress protein) and Dam. Absence of methylation within GATC sites of the antigen 43 (*agn*43) gene promoter region has been reported to lead to lowered growth rates and may have influenced outcomes of biofilm formation assays for this study (90, 91).

Biofilm formation contributes to the *in vivo* survival and persistence of UPEC during UTI and plays a pivotal role in the colonization of the urogenital tract (92). The production of oxidative radicals by uroepithelial cells have been reported to enhance UPEC fitness by the induction of DNA repair systems to combat proceeding exposures, subsequent to infection – a characteristic critical for virulence (93). Interestingly, after 24 h of exposure *dam* mutants strains displayed greater persistence rates (higher cell density) but variable responses to increases in reactive oxygen species. This may be attributed to spontaneous mutation frequencies among such strains since the oxidative stress response proteins OxyR and SoxRS (principal regulators for hydrogen peroxide detoxification) in *E. coli* are competitive to Dam for overlapping GATC sites in, or near promoters (94). Additionally, the negative regulation of the antigen 43 phase variable biofilm formation autotransporter boosts autoaggregation and 3D appearance (94). In the absence of *dam*, the phase variation protein binding (epigenetic switch) at three GATC sites in *agn*43 (*flu*) is hampered since activation occurs subsequent to methylation (34). Contrary to expected outcomes, this should lower cell aggregation among mutant cells. However, the augmented occurrence of stress-induced mutagenesis and repair activated by upregulated SOS gene expression may account for elevated levels of biofilm formation among UPEC *dam* mutants, contrary to at least one report of increased sensitivity among *E. coli dam* mutants by Calmann and Marinus (95).

Further antimicrobial susceptibility studies conducted on cured and pGEMQA C119 parental and *dam-*mutant strains as well as pGEMQA transformants of CFT073 wild type and *dam-*mutant strains indicated variable phenotypic responses. Likewise, the capricious observation for *dam-*mutant strains (further enhanced with the presence of pGEMQA by up to threefold that of parental and *dam* complement strains) may have been a result of SOS activation triggered by quinolones inhibiting the topoisomerase IV activity (72, 82). Dam-mutant strains of C119 were notably altered in resistance against various classes of antimicrobial agents by as much as eightfold in comparison to parental strains. Such variability displayed among mutants could be attributed to increased mutation rates and accumulating secondary mutations during growth. The latter would be of significant survival value in the presence of increased SOS response activators (oxidative and DNA damaging stress) and for the purpose of increased biofilm structure production.

The contribution of Dam methylation to the expression and adherence of virulence factor type 1 fimbriae in UPEC to host cells is well known to have a perceptible effect (38). Equally, Dam’s role in the epigenetic switch of P fimbriae is of significance to the pathogenicity of uropathogenic *E. coli* strains implicated in UTIs. Transcriptionally controlled by cooperative binding of the leucine-responsive regulatory protein (Lrp) and translocation by ancillary protein PapI, the Pap phase variation is dependent on Dam methylation along the GATC sites upstream the *pap*BA-regulatory region (80). However, it was not until Campellone et al. (26) reported on the increased adherence of EHEC *dam* mutants to cultured mammalian cells when compared to wild-type EHEC that it was clear the absence of Dam function somehow triggered alternate virulence pathways; in this case, negatively regulating binding to host cells. Similarly, we make the first report of this phenomenon by UPEC Δ*dam* strains compared to wild-type strains with the preferred adherence of mutant strains to cultured HTB-9 bladder cells. While it is clear that there are differences in attachment between *dam* mutant and wild-type strains, it does not preclude the involvement of other uroadhesion mechanisms which share regulatory features to *pap*, e.g., afimbrial adhesin, and Type 1 and S fimbriae (96). Moreover, comparable to previous findings (25), the subtle adherence exhibited by UPEC strains to cell culture models (post-1.5 h) coincides with discrepancies noted since up to 40% of UPEC isolates display no adherence to uroepithelial cells, are almost always afimbriated and thus, may not adhere when freshly isolated or cultured (7).

Furthermore, semi-quantitative RT-PCR results also reflected this variation in gene expression as there was a measurable increase in the transcription levels of the *pap*I gene of Δ*dam* versus wild-type bacteria, suggesting that the increase in fimbriae is a direct result of alteration to the transcription initiation at regulatory sites. In this case, the alteration may have been the lack of *dam* methylation pattern control at both distal and proximal sites within the *pap* regulatory DNA leading to a constant phase ON switch for expression of the operon; a finding consistent with the report by Marinus and Casedesús (34). It is hypothesized that the reversible expression of *pap* allows the bacteria to attach and detach from the urogenital tissues as needed. In theory this enables colonization and infection of the bladder, then subsequently the kidney, leading to cystitis and pyelonephritis, respectively (33). However, the preferential attachment of the UPEC strains to bladder cells (seen in this study) may indicate either the occurrence of variation in PapG adhesin specificity to globoseries of the tubular mucosal cells, or the associated level of pathogenicity potential for causing infection in these tissues (17, 20, 21).

Notably, the increased expression of *rec*A, *pap*I, and *qnr*A but decreased or absent expression of *dam, mdh, pap*EF, *arc*A, *gyr*B observed among *dam* mutant and *dam*-complement strains comparative to wild-types, underscores the high level of altered phenotypes present as a result of altered gene expression. Numerous studies with RecA have indicated its role as chief regulator in SOS induction, homologous recombination mechanisms and eventual phenotypic variations by means of phenotypic switching (72, 74, 88). In the presence of environmental changes (e.g., oxidative stress, DNA damage), the interplay between *dam* and *rec*A may be integral to the altered responses of cells for adaptation. Evidence points to increased minimal level of expression of *rec*A in *dam* mutants relative to wild-type strains (88, 97). Various studies have shown the full functional capability of methyl-directed mismatch repair in *E. coli dam* mutants to induce increased nucleotide base substitutions and mutagenic double-stranded breaks (72, 88). Such occurrences contribute to the increased mutability and hypervariation demonstrated by mutant strains (87, 98). Moreover, excessive recombinational events and insufficient repair of chromosome breaks in *dam* mutants eventually leads to cell death as shown by the non-viability of double mutants of *dam rec*A, *dam ruv*A, *dam pol*A, *dam lig*, and *dam pri*A (76, 88).

The preferential expression and modulation of several virulence genes by *dam* mutants (relative to wild-type) of pathogenic *Salmonella* strains (36) advocates the therapeutic potential of attenuated strains as live vaccines (33, 36, 47) and Dam inhibitors as antibacterial drugs (38, 46). Despite this background, our findings illustrating sustained viability and low level of adherence *in vitro* to uroepithelial cells by Δ*dam* UPEC strains may dampen the prospect for solitary use of Dam inhibitors or UPEC Δ*dam* strains as live vaccines. Further characterization and post-transcriptional and *in vivo* investigations for the influence of DNA adenine methylation on virulence gene regulation may reveal associated regulatory networks and their value to vital functions indispensable to pathogenicity of UPEC and other bacteria.

## Author Contributions

SA-MS participated in all experiments and designing of the research plan, coordinated the data analysis and contributed to the writing of the manuscript, approved the final version, and agreed to be accountable for all aspects of the work. PB conceived and organized the study, designed the research plan and contributed to the writing of the manuscript, approved the final version, and agreed to be accountable for all aspects of the work.

## Conflict of Interest Statement

The authors declare that the research was conducted in the absence of any commercial or financial relationships that could be construed as a potential conflict of interest.

## References

[1] Welch RA Burland V Plunkett G Redford P Roesch P Rasko D Extensive mosaic structure revealed by the complete genome sequence of uropathogenic *Escherichia coli*. Proc Natl Acad Sci U S A (2002) 99(26):17020–4. 10.1073/pnas.252529799 12471157 PMC139262

[2] Zalmanovici Trestioreanu A Green H Paul M Yaphe J Leibovici L. Antimicrobial agents for treating uncomplicated urinary tract infection in women. Cochrane Database Syst Rev (2010) 10:CD007182. 10.1002/14651858 20927755 PMC12501835

[3] Maroncle NM Sivick KE Brady R Stokes F-E Mobley HLT. Protease activity, secretion, cell entry, cytotoxicity and cellular targets of secreted autotransporter toxin of uropathogenic *Escherichia coli*. Infect Immun (2006) 74:6124–34. 10.1128/IAI.01086-06 16954394 PMC1695523

[4] Salyers AA Whitt DD Bacterial Pathogenesis: A Molecular Approach. 2nd ed Washington, DC: ASM Press (2002).

[5] Li K Zhou W Hong Y Sacks SH Sheerin NS Synergy between type I fimbriae expression and C3 opsonization increases internalisation of *E. coli* by human tubular epithelial cells. BMC Microbiol (2009) 9:64 10.1186/1471-2180-9-64 19335887 PMC2670304

[6] Johnson JR Stell AL. Extended virulence genotypes of *Escherichia coli* strains from patients with urosepsis in relation to phylogeny and host compromise. J Infect Dis (2000) 18:261–72. 10.1086/315217 10608775

[7] Miyazaki J Ba-Thein W Kumao T Yasuoka MO Asaka H Hayshi H. Type 1, P and S fimbriae, and afimbrial adhesin I are not essential for uropathogenic *Escherichia coli* to adhere to and invade bladder epithelial cells. FEMS Immunol Med Microbiol (2002) 33:23–6. 10.1111/j.1574-695X.2002.tb00567.x 11985964

[8] Blum G Falbo V Caprioli A Hacker J Gene clusters encoding the cytotoxic necrotizing factor type I, Prs-fimbriae and α-haemolysin form the pathogenicity island II of the uropathogenic *Escherichia coli* strain J96. FEMS Microbiol Lett (1995) 126:189–96. 10.1016/0378-1097(95)00009-T 7705611

[9] Hacker J Genetic determinants coding for fimbriae and adhesins of extraintestinal *Escherichia coli*. Curr Top Microbiol Immunol (1990) 151:1–27. 1973366 10.1007/978-3-642-74703-8_1

[10] High NJ Hales BA Jann K Boulnois GJ A block of urovirulence genes encoding multiple fimbriae and haemolysin in *Escherichia coli* O4:K12:H^-^. Infect Immun (1988) 56:513–7. 2892797 10.1128/iai.56.2.513-517.1988 PMC259312

[11] Kao J-S Stucker DM Warren JW Mobley HL. Pathogenicity island sequences of pyelonephritogenic *Escherichia coli* CFT073 are associated with virulent uropathogenic strains. Infect Immun (1997) 65:2812–20. 9199454 10.1128/iai.65.7.2812-2820.1997 PMC175396

[12] Knapp S Hacker J Jarchau T Goebel W. Large, unstable inserts in the chromosome affect virulence properties of uropathogenic *Escherichia coli* O6 strain 536. J Bacteriol (1986) 168:22–30. 2875989 10.1128/jb.168.1.22-30.1986 PMC213415

[13] Low D David V Lark D Schoolnik G Falkow S Gene clusters governing the production of haemolysin and mannose-resistant haemagglutination are closely linked in *Escherichia coli* serotype O4 and O6 isolates from urinary tract infections. Infect Immun (1984) 43:353–8. 6317570 10.1128/iai.43.1.353-358.1984 PMC263434

[14] Swenson DL Bukanov NO Berg DE Welch RA. Two pathogenicity islands in uropathogenic *Escherichia coli* J96: cosmid cloning and sample sequencing. Infect Immun (1996) 64:3736–43. 8751923 10.1128/iai.64.9.3736-3743.1996 PMC174287

[15] Wullt B Erratum to “the role of P fimbriae for *Escherichia coli* establishment and mucosal inflammation in the human urinary tract” [Int J Antimicrob Agents 19 (2002): 522-538]. Int J Antimicrob Agents (2003) 21(6):605–21. 10.1016/S0924-8579(02)00103-6 13678032

[16] Eto DS Jones TA Sundsbak JL Mulvey MA Integrin-mediated hostcell invasion by type I-piliated uropathogenic *Escherichia coli*. PLoS Pathog (2007) 3:e100 10.1371/journal.ppat.0030100 17630833 PMC1914067

[17] Godaly G Svanborg C. Urinary tract infections revisited. Kidney Int (2007) 71:721–3. 10.1038/sj.ki.5002170 17429419

[18] Hoepelman AIM Tuomanen EI. Consequences of microbial attachment: directing host cell functions with adhesins. Infect Immun (1992) 60:1729–33. 1563758 10.1128/iai.60.5.1729-1733.1992 PMC257065

[19] Johnson JR. Virulence factors in *Escherichia coli* urinary tract infection. Clin Microbiol Rev (1991) 4:80–128. 10.1128/CMR.4.1.80 1672263 PMC358180

[20] Melican K Sandoval RM Kader A Josefsson L Tanner GA Molitons BA Uropathogenic *Escherichia coli* P and type I fimbriae act in synergy in a living host to facilitate renal colonization leading to nephron obstruction. PLoS Pathog (2011) 7:e100298 10.1371/journal.ppat.1001298 PMC3044688 21383970

[21] Wright KJ Hultgren SJ. Sticky fibers and uropathogenesis: bacterial adhesins in the urinary tract. Future Microbiol (2006) 1:75–87. 10.2217/17460913.1.1.75 17661687

[22] Holden NJ Gally DL Switches, cross-talks and memory in *Escherichia coli* adherence. J Med Microbiol (2004) 53:585–93. 10.1099/jmm.0.05491-0 15184527

[23] Hagberg L Jodal U Korhonen TK Lidin-Janson G Lindberg U Svanborg Eden C Adhesion, haemagglutination, and virulence of *Escherichia coli* causing urinary tract infections. Infect Immun (1981) 31:564–70. 7012012 10.1128/iai.31.2.564-570.1981 PMC351345

[24] Kreft B Placzek M Dehn C Hacker J Schmidt G Waseunauer G S fimbriae of uropathogenic *Escherichia coli* binding to primary human renal proximal tubular epithelial cells do not induce expression of intercellular adhesion molecule 1. Infect Immun (1995) 63:3235–8. 7622256 10.1128/iai.63.8.3235-3238.1995 PMC173445

[25] Lane MC Mobley HL Role of P-fimbrial mediated adherence in pyelonephritis and persistence of uropathogenic *Escherichia coli* (UPEC) in the mammalian kidney. Kidney Int (2007) 72:19–25. 10.1038/sj.ki.5002230 17396114

[26] Campellone KG Rose AJ Løbner-Olesen A Murphy KC Magoun L Brady MJ Increased adherence and catin pedestal formation by *dam*-deficient enterohaemorrhagic *Escherichia coli* O157:H7. Mol Microbiol (2007) 63:1468–81. 10.1111/j.1365-2958.2007.05602.x 17302821

[27] Chen YT Shu HY Li LH Liao TL Wu KM Shiau YR Complete nucleotide sequence of pK245 a 98-kilobase plasmid conferring quinolone resistance and extended-spectrum-β-lactamase activity in a clinical *Klebsiella pneumoniae* isolate. Antimicrob Agents Chemother (2006) 50:3861–6. 10.1128/AAC.00456-06 16940067 PMC1635178

[28] Erova TE Pillai L Fadl AA Sha J Wang S Galindo CL DNA adenine methyltransferase influences the virulence of *Aeromonas hydrophila*. Infect Immun (2006) 74:410–24. 10.1128/IAI.74.1.410-424.2006 16368997 PMC1346675

[29] Løbner-Olesen A Skøvgaard O Marinus MG. *Dam* methylation: coordinating cellular processes. Curr Opin Microbiol (2005) 8:154–60. 10.1016/j.mib.2005.02.009 15802246

[30] Low DA Weyand NJ Mahan MJ Roles of DNA adenine methylation in regulating bacterial expression and virulence. Infect Immun (2001) 69:7197–204. 10.1128/IAI.69.12.7197-7204.2001 11705888 PMC98802

[31] Fälker S Schmidt MA Heusipp G DNA methylation in *Yersinia enterocolitica*: role of DNA adenine methyltransferase in mismatch repair and regulation of virulence factors. Microbiology (2005) 151:2291–9. 10.1099/mic.0.27946-0 16000719

[32] Ostendorf T Cherepanov P De Vries J Wackernagel W Characterization of a *dam* mutant of *Serratia marescens* and nucleotide sequence of the *dam* region. J Bacteriol (1999) 181:3880–5. 10383952 10.1128/jb.181.13.3880-3885.1999 PMC93874

[33] Julio SM Heithoff DM Provenzano D Klose KE Sinsheimer RL Low DA DNA adenine methylase is essential for viability and plays a role in the pathogenesis of *Yersinia pseudotuberculosis* and *Vibrio cholera*. Infect Immun (2001) 69:7610–5. 10.1128/IAI.69.12.7610-7615.2001 11705940 PMC98854

[34] Marinus MG Casadesús J. Roles of DNA adenine methylation in host-pathogen interactions: mismatch, repair, transcriptional regulation, and more. FEMS Microbiol Rev (2009) 33:488–503. 10.1111/j.1574-6976.2008.00159.x 19175412 PMC2941194

[35] Reisenauer A Kahng LS McCollum S Shapiro L Bacterial DNA methylation: a cell cycle regulator? J Bacteriol (1999) 181:5135–9. 10464180 10.1128/jb.181.17.5135-5139.1999 PMC94015

[36] Badie G Heithoff DM Sinsheimer RL Mahan MJ Altered levels of *Salmonella* DNA adenine methylase are associated with defects in gene expression, motility, flagellar synthesis, and bile resistance in the pathogenic strain 14028 but not in laboratory strain LT2. J Bacteriol (2007) 189:1556–64. 10.1128/JB.01580-06 17172341 PMC1855711

[37] Braaten BA Platko JV van der Woude MW Simons BH de Graaf FK Calvo JM Leucine-responsive regulatory protein controls the expression of both the *pap* and *fan* pili operons in *Escherichia coli*. Proc Natl Acad Sci U S A (1992) 9:4250–4. 10.1073/pnas.89.10.4250 1350087 PMC49059

[38] Casadesús J Low D. Epigenetic gene regulation in the bacterial world. Microbiol Mol Biol Rev (2006) 70:830–56. 10.1128/MMBR.00016-06 16959970 PMC1594586

[39] García Del-Portillo F Pucciarelli MG Casadesús J DNA adenine methylase mutants of *Salmonella typhimurium* show defects in protein secretion, cell invasion, and M cell cytoxicity. Proc Natl Acad Sci U S A (1999) 96:1178–83. 10.1073/pnas.96.20.11578 PMC18076 10500219

[40] Hernday AD Braaten BA Low DA. The mechanism by which DNA adenine methylase and *Pap*I activate the *pap* epigenetic switch. Mol Cell (2003) 12:947–57. 10.1016/S1097-2765(03)00383-6 14580345

[41] Blyn LB Braaten BA Low DA. Regulation of *pap* pilin phase variation by a mechanism involving differential *dam* methylation states. EMBO J (1990) 9:4045–54. 2147413 10.1002/j.1460-2075.1990.tb07626.x PMC552177

[42] Weyand NJ Low DA. Regulation of Pap phase variation. Lrp is sufficient for the establishment of the phase OFF *pap* DNA methylation pattern and repression of *pap* transcription *in vitro*. J Biol Chem (2000) 275:3192–200. 10.1074/jbc.275.5.3192 10652304

[43] van Der Woude MW Braaten BA Low DA Evidence for a global regulatory control of pilus expression in *Escherichia coli* by Lrp and DNA methylation: model building based on analysis of *pap*. Mol Microbiol (1992) 6:2429–35. 10.1111/j.1365-2958.1992.tb01418.x 1357527

[44] van der Velden AW Baumler AJ Tsolis RM Heffron F. Multiple fimbrial adhesins are required for full virulence of *Salmonella typhimurium* in mice. Infect Immun (1998) 66:2803–8. 9596751 10.1128/iai.66.6.2803-2808.1998 PMC108273

[45] Heithoff DM Badie G Julio SM Enioutina EY Daynes RA Sinsheimer RL *In vivo*-selected mutations in methyl-directed mismatch repair suppress the virulence attenuation of *Salmonella dam* mutant strains following intraperitoneal, but not oral, infection of naïve mice. J Bacteriol (2007) 189(13):4708–17. 10.1128/JB.00299-07 17468250 PMC1913454

[46] Jakomin M Chessa D Bäumler AJ Casadesús J Regulation of the *Salmonella enteric std* fimbrial operon by DNA adenine methylation, SeqA, and HdfR. J Bacteriol (2008) 190:7406–13. 10.1128/JB.01136-08 18805972 PMC2576648

[47] Plumbridge J The role of *dam* methylation in controlling gene expression. Biochimie (1987) 69:439–43. 10.1016/0300-9084(87)90081-2 3118961

[48] Stephenson S Brown PD Holness A Wilks M. The emergence of *qnr*-mediated quinolone resistance among Enterobacteriaceae in Jamaica. West Indian Med J (2010) 59:241–4. 21291099

[49] Stephenson S Brown PD Occurrence of class I integrons among uropathogenic fluoroquinolone-resistant *Escherichia coli* isolates in Jamaica. APMIS (2013) 121(3):226–31. 10.1111/j.1600-0463.2012.02960.x 23030058

[50] Mobley HL Green DM Trifillis AL Johnson DE Chippendale GR Lockatell CV, editors. Pyelonephritogenic *Escherichia coli* and killing of cultured human renal proximal tubular epithelial cells: role of hemolysin in some strains. Infect Immun (1990) 58:1281–9. 2182540 10.1128/iai.58.5.1281-1289.1990 PMC258621

[51] Murphy KC Campellone KG. Lambda Red-mediated recombinogenic engineering of enterohemorrhagic and enteropathogenic *E. coli*. BMC Mol Biol (2003) 4:11. 10.1186/1471-2199-4-11 14672541 PMC317293

[52] Datsenko KA Wanner BL. One-step inactivation of chromosomal genes in *Escherichia coli* K-12 using PCR products. Proc Natl Acad Sci U S A (2000) 97:6640–5. 10.1073/pnas.120163297 10829079 PMC18686

[53] Blattner FR Plunkett G Bloch CA Perna NT Burland V Riley M The complete genome sequence of *Escherichia coli* K-12. Science (1997) 277:1453–74. 10.1126/science.277.5331.1453 9278503

[54] McDonald LC Chen F-J Lo H-J Yin H-C Lu P-L Huang C-H Emergence of reduced susceptibility and resistance to fluoroquinolones in *Escherichia coli* in Taiwan and contributions of distinct selective pressures. Antimicrob Agents Chemother (2001) 45(11):3084–91. 10.1128/AAC.45.11.3084-3091.2001 11600360 PMC90786

[55] Komp Lindgren P Karlsson Å Hughes D. Mutation rate and evolution of fluoroquinolone resistance in *Escherichia coli* isolates from patients with urinary tract infections. Antimicrob Agents Chemother (2003) 47(10):3222–32. 14506034 10.1128/AAC.47.10.3222-3232.2003 PMC201150

[56] Rodriguez-Siek KE Giddings CW Doetkott C Johnson TJ Fakhr MK Nolan LK Comparison of *Escherichia coli* isolates implicated in human urinary tract infection and avian colibacillosis. Microbiology (2005) 151:2097–110. 10.1099/mic.0.27499-0 15942016

[57] Beutin L Strauch E Zimmermann S Kaulfuss S Schaudinn C Männel A Genetical and functional investigation of *fliC* genes encoding flagellar serotype H4 in wildtype strains of *Escherichia coli* and in a laboratory *E. coli* K-12 strain expressing flagellar antigen type H48. BMC Microbiol (2005) 5:4. 10.1186/1471-2180-5-4 15663798 PMC548302

[58] Wirth T Falush D Lan R Colles F Mensa P Wieler LH Sex and virulence in *Escherichia coli*: an evolutionary perspective. Mol Microbiol (2006) 60(5):1136–51. 10.1111/j.1365-2958.2006.05172.x 16689791 PMC1557465

[59] Ferreira A Rendano L Wiedmann M Boor KJ. Characterization of rpoS alleles in *Escherichia coli* O157:H7 and in other *E. coli* serotypes. J Appl Microbiol (1999) 86:295–301. 10.1046/j.1365-2672.1999.00664.x 10063629

[60] Robicsek A Jacoby GA Hooper DC. The worldwide emergence of plasmid mediated quinolone resistance. Lancet Infect Dis (2006) 6:629–40. 10.1016/S1473-3099(06)70599-0 17008172

[61] Chen L Paulsen DB Scruggs DW Banes MM Reeks BY Lawrence ML. Alteration of DNA adenine methylase (*Dam*) activity in *Pasteurella multocida* causes increased spontaneous mutation frequency and attenuation in mice. Microbiology (2003) 149:2283–90. 10.1099/mic.0.26251-0 12904568

[62] Matlock BC Beringer RW Ash DL Page AF Differences in Bacterial Optical Density Measurements between Spectrophotometers. Thermo Fisher Scientific Technical Note 52236. Madison, WI: Thermo Fisher Scientific (2011).

[63] Chapin KC Musgnug MC. Validation of the automated reading and incubation system with Sensititre plates for antimicrobial susceptibility testing. J Clin Microbiol (2003) 41:1951–6. 10.1128/JCM.41.5.1951-1956.2003 12734233 PMC154729

[64] Hedge A Bhat GK Mallya S. Effect of exposure to hydrogen peroxide on the virulence of *Escherichia coli*. Indian J Med Microbiol (2008) 26(1):25–8. 10.4103/0255-0857.38853 18227593

[65] Hryckowian AJ Welch RA RpoS contributes to resistance to phagocyte oxidase-mediated stress during urinary tract infection by *Escherichia coli* CFT073. mBio (2013) 4(1):e23–13. 10.1128/mBio.00023-13 PMC3573659 23404396

[66] Marone M Mozzetti S De Ritis D Pierelli L Scambia G. Semiquantitative RT-PCR analysis to assess the expression levels of multiple transcripts from the same sample. Biol Proced Online (2001) 3:19–25. 10.1251/bpo20 12734582 PMC145543

[67] Holden N Totsika M Dixon L Catherwood K Gally DL. Regulation of P-fimbrial phase variation frequencies in *Escherichia coli* CFT073. Infect Immun (2007) 75:3325–34. 10.1128/IAI.01989-06 17452474 PMC1932927

[68] Volkmer B Heinemann M. Condition-dependent cell volume and concentration of *Escherichia coli* to facilitate data conversion for systems biology modeling. PLoS One (2011) 6(7):e23126. 10.1371/journal.pone.0023126 21829590 PMC3146540

[69] Marinus MG. DNA methylation and mutator genes in *Escherichia coli* K-12. Mutat Res (2010) 705:71–6. 10.1016/j.mrrev.2010.05.001 20471491 PMC2932865

[70] Heitman J Model P. Site-specific methylases induce the SOS DNA repair response in *Escherichia coli*. J Bacteriol (1987) 169(7):3243–50. 3036779 10.1128/jb.169.7.3243-3250.1987 PMC212376

[71] Justice SS Hunstad DA Seed PC Hultgren SJ. Filamentation by *Escherichia coli* subverts innate defenses during urinary tract infection. Proc Natl Acad Sci U S A (2006) 103(52):19884–9. 10.1073/pnas.0606329104 17172451 PMC1750882

[72] Janion C Some aspects of the SOS response system – a critical survey. Acta Biochim Pol (2001) 48(3):599–610. 11833768

[73] O’Reilly EK Kreuzer KN. Isolation of SOS constitutive mutants of *Escherichia coli*. J Bacteriol (2004) 186(21):7149–60. 10.1128/JB.186.21.7149-7160.2004 15489426 PMC523190

[74] Janion C. Inducible SOS response system of DNA repair and mutagenesis in *Escherichia coli*. Int J Biol Sci (2008) 4(6):338–44. 10.7150/ijbs.4.338 18825275 PMC2556049

[75] Riva A Delorme M-O Chevalier T Guilhot N Hénaut C Hénaut A. Characterization of the GATC regulatory network in *E. coli*. BMC Genomics (2004) 5:48. 10.1186/1471-2164-5-48 15265237 PMC493266

[76] Robbins-Manke JL Zdraveski ZZ Marinus M Essigmann JM. Analysis of global gene expression and double-strand-break formation in DNA adenine methyltransferase- and mismatch repair-deficient *Escherichia coli*. J Bacteriol (2005) 187(20):7027–37. 10.1128/JB.187.20.7027-7037.2005 16199573 PMC1251628

[77] Kamenšek S Podlesek Z Gillor O Žgur-Bertok D Genes regulated by the *Escherichia coli* SOS repressor LexA exhibit heterogenous expression. BMC Microbiol (2010) 10:283 10.1186/1471-2180-10-283 21070632 PMC2994835

[78] Wu H Lippmann JE Oza JP Zeng M Fives-Taylor P Reich NO. Inactivation of DNA adenine methyltransferase alters virulence factors in *Actinobacillus actinomycetemcomitans*. Oral Microbiol Immunol (2006) 21:238–44. 10.1111/j.1399-302X.2006.00284.x 16842508

[79] Marinus MG Methylation of DNA. In: Neidhardt FC Curtis R III Ingraham JL Lin ECC Low KB Magasanik B Escherichia coli and Salmonella: Cellular and Molecular Biology. 2nd edn Washington, DC: ASM Press (1996). p. 782–91.

[80] Wion D Casadesus J. N6-methyl-adenine: an epigenetic signal for DNA-protein interactions. Nat Rev Microbiol (2006) 4:183–92. 10.1038/nrmicro1350 16489347 PMC2755769

[81] Boye E Løbner-Olesen A Skarstad K. Timing of chromosomal replication in *Escherichia coli*. Biochim Biophys Acta (1988) 951(2–3):359–64. 10.1016/0167-4781(88)90107-8 2850013

[82] Palmer BR Marinus MG The *dam* and *dcm* strains of *Escherichia coli* – a review. Gene (1994) 143:1–12. 10.1016/0378-1119(94)90597-5 8200522

[83] Marinus MG Løbner-Olesen A DNA methylation. EcoSal Plus (2014) 6(1). 10.1128/ecosalplus.ESP-0003-2013 PMC4231299 26442938

[84] Løbner-Olesen A Marinus MG Hansen FG. Role of SeqA and Dam in *Escherichia coli* gene expression: a global/microarray analysis. Proc Natl Acad Sci USA (2003) 100(8):4672–7. 10.1073/pnas.0538053100 12682301 PMC153614

[85] Rasmussen LJ Marinus MG Løbner-Olesen A. Novel growth rate control of dam gene expression in *Escherichia coli*. Mol Microbiol (1994) 12:631–8. 10.1111/j.1365-2958.1994.tb01050.x 7934887

[86] Marinus MG Morris NR Biological function for 6-methyladenine residues in the DNA of *Escherichia coli* K12. J Mol Biol (1974) 85:309–22. 10.1016/0022-2836(74)90366-0 4600143

[87] Ponder RG Fonville NC Rosenberg SM. A switch from high-fidelity to error-prone DNA double-strand break repair underlies stress-induced mutation. Mol Cell (2005) 19:791–804. 10.1016/j.molcel.2005.07.025 16168374

[88] Marinus MG. Recombination is essential for viability of an *Escherichia coli dam* (DNA adenine methyltransferase) mutant. J Bacteriol (2000) 182(2) 463–8. 10.1128/JB.182.2.463-468.2000 10629194 PMC94297

[89] Zaleski P Piekarowicz A. Characterization of a *dam* mutant of *Haemophilus influenzae* Rd. Microbiology (2004) 150:3773–81. 10.1099/mic.0.27225-0 15528663

[90] Lim HN van Oudenaarden A A multistep epigenetic switch enable the stable inheritance of DNA methylation states. Nat Genet (2007) 39:269–75. 10.1038/ng1956 17220888

[91] Wallecha A Corventi J Munster V van der Woude M. Phase variation of Ag43 is independent of the oxidation state of OxyR. J Bacteriol (2003) 185:2203–9. 10.1128/JB.185.7.2203-2209.2003 12644490 PMC151510

[92] Costerton JW Stewart PS Greenberg EP. Bacterial biofilms: a common cause of persistent infections. Science (1999) 284(5418):1318–22. 10.1126/science.284.5418.1318 10334980

[93] Li B Smith P Horvath DJ Jr Romesberg FE Justice SS. SOS regulatory elements are essential for UPEC pathogenesis. Microbes Infect (2010) 12(8–9):662–8. 10.1016/j.micinf.2010.04.009 20435157

[94] Chiang SM Schellhorn HE. Regulators of oxidative stress response genes in *Escherichia coli* and their functional conservation in bacteria. Arch Biochem Biophys (2012) 525(2):161–9. 10.1016/j.abb.2012.02.007 22381957

[95] Calmann MA Marinus MG. Regulated expression of the *Escherichia coli dam* gene. J Bacteriol (2003) 185:5012–4. 10.1128/JB.185.16.5012-5014.2003 12897023 PMC166483

[96] Giron JA Torres AG Freer E Kaper JB. The flagella of enteropathogenic *Escherichia coli* mediate adherence to epithelial cells. Mol Microbiol (2002) 44:361–79. 10.1046/j.1365-2958.2002.02899.x 11972776

[97] Peterson KR Wertman KF Mount DW Marinus MG Viability of *Escherichia coli* K-12 DNA adenine methylase (*dam*) mutants requires increased expression of sp in the SOS regulon. Mol Genet Genomics (1985) 201:14–9. 10.1007/BF00397979 3932821

[98] Wyrzykowski J Volkert MR. The *Escherichia coli* methyl-directed mismatch repair system repairs base pairs containing oxidative lesions. J Bacteriol (2003) 185(5):1701–4. 10.1128/JB.185.5.1701-1704.2003 12591888 PMC148063

